# Comment on “Nationwide randomised trial evaluating elective neck dissection for early-stage oral cancer (SEND study) with meta-analysis and concurrent real-world cohort.”

**DOI:** 10.1038/s41416-020-0981-9

**Published:** 2020-07-16

**Authors:** Harsh Dhar, Richa Vaish, Anil K. D’Cruz

**Affiliations:** 1 Department of Head and Neck Oncology, Narayana Superspeciality Hospitals, Howrah, West Bengal 711103 India; 2 Department of Head and Neck Oncology, 1. Tata Memorial Hospital 2. Homi Bhabha National Institute, Mumbai, 400012 India; 3 Director Oncology, Apollo Hospitals, Navi Mumbai, 400614 India; 4 Union International Cancer Control (UICC), Geneva, Switzerland; 5 Ex-Director and Chief of Head Neck Services, Tata Memorial Hospital, Mumbai, 4000012 India

**Keywords:** Surgical oncology, Oral cancer

Sir,

We read with great interest the results of the randomised trial and meta-analysis conducted by Hutchison et al. showing the benefit of Elective Neck Dissection (END) in early oral cancers that was published in your esteemed journal (volume 121)^1^ and congratulate the authors.

Results of this trial reinforced the benefit of electively treating the neck over a wait and watch policy. Despite not having completed accrual the trial was prematurely terminated subsequent to the publication of the results of the RCT from our institution.^2^ It is noteworthy that despite smaller numbers there was a clear benefit of electively treating the neck in terms of DFS and locoregional recurrence and a trend towards OS benefit (which in all probability would have reached significance had the trial completed accrual). In addition, the study demonstrates that quality of life scores across both treatment arms were highly similar, extending greater credibility to the safety of a well-performed elective neck dissection.

Moreover, the cost-effectiveness analysis indicates that performing an END obviates the cost of a more extensive therapeutic neck dissection and adjuvant therapy that patients may have to undergo later.

While there were differences in patient demography between the SEND study and our trial, the cohorts were not grossly dissimilar given that majority of patients in both trials had tumours of the oral tongue and median depth of invasion in both was comparable (4.5 and 5 mm in the 2 arms of SEND trial and 6 mm in the TMH Mumbai trial). The marginally higher value of median depth in the latter can be explained by the SEND trial including lesions with a maximal T size up to 3 cm compared to 4 cm.

Another pertinent finding of clinical relevance from the SEND trial is the benefit of END evident in the T1 subgroup, which was sustained even after eliminating the thicker tumours based on revised AJCC. Analysis of the patients with depth ≤ 3 mm indicated better DFS with END. This adds to the results of the TMH Mumbai trial in which END offered benefit for all tumours with DOI > 3 mm irrespective of size, strongly supporting the need for END in thin T1 lesions as well. This finding is at variance with recent ASCO guidelines,^3^ which did recommend the option of wait and watch in patients with thinner tumours who are reliable for follow up.

Given that the study was conducted in 25 institutions across the UK, we agree with the authors that it is representative of a wider community-based practice.

We are compelled to write this letter so late after the publication of the SEND trial because literature is still flooded with publications and meta-analysis^4–10^ despite the highest form of evidence by way of two large randomised trials in favour of elective neck dissection. One of these meta-analyses (Ren et al.)^4^ even commented that there was no need for more randomised trials. We believe that publication of this letter will yet again decisively convey to the Head and Neck oncologists of the benefit of END and motivate them to spend time and resources on more compelling issues such as the role of Sentinel Node biopsy in lieu of neck dissection. We stand the risk of subjecting our patients to poorer disease control by observing the neck in spite of having irrefutable evidence in favour of END.

## Data Availability

This is a correspondence to an earlier published article in BJC, which has been mentioned along with other supporting articles in the references section.
